# Navigating Intraductal Papillary Mucinous Neoplasm Management through Fukuoka Consensus vs. European Evidence-Based Guidelines on Pancreatic Cystic Neoplasms—A Study on Two European Centers

**DOI:** 10.3390/cancers16112156

**Published:** 2024-06-06

**Authors:** Vladimir Djordjevic, Djordje Knezevic, Blaz Trotovsek, Ales Tomazic, Miha Petric, Benjamin Hadzialjevic, Nikica Grubor, Mihajlo Djokic

**Affiliations:** 1 First Surgical Clinic, University Clinical Center of Serbia, 11000 Belgrade, Serbia; djordje.knezevic@kcs.ac.rs (D.K.); nikica.grubor@kcs.ac.rs (N.G.); 2 University Medical Center Ljubljana, University of Ljubljana, 1000 Ljubljana, Slovenia; blaz.trotovsek@kclj.si (B.T.); ales.tomazic@kclj.si (A.T.); miha.petric@kclj.si (M.P.); benjamin.hadzialjevic@kclj.si (B.H.); mihajlo.djokic@kclj.si (M.D.)

**Keywords:** pancreatic cystic neoplasm, IPMN, guidelines, cancer, management, Serbia, Slovenia

## Abstract

**Simple Summary:**

This research delves into the realm of pancreatic cystic neoplasms (PCNs), specifically focusing on a subtype with a high malignancy potential that is known as intraductal papillary mucinous neoplasms (IPMNs). By analyzing data from 113 patients across two European medical centers, this study assesses the effectiveness of two key guidelines, the Fukuoka consensus guidelines and the European evidence-based guidelines, in diagnosing severe disease stages in IPMNs. The findings reveal comparable diagnostic accuracies between the guidelines, highlighting the importance of personalized patient care and the potential indicators for surgical intervention. This study underscores the need for continuous research to refine these guidelines to improve patient outcomes and deepen our understanding of PCNs. Through this research, the authors seek to contribute to more accurate diagnosis and treatment strategies, ultimately influencing the broader medical community’s approach to managing these complex cystic formations in the pancreas.

**Abstract:**

This study addresses the critical need for the accurate diagnosis and management of intraductal papillary mucinous neoplasms (IPMNs), which are pancreatic cystic neoplasm types holding a substantial potential for malignancy. It evaluates the diagnostic effectiveness of the Fukuoka consensus guidelines and the European evidence-based guidelines in detecting high-grade dysplasia/invasive carcinoma in IPMNs, utilizing a retrospective analysis of 113 patients from two European medical centers. The methods include a comparative analysis of clinical, radiological, and endoscopic ultrasonography data, alongside an assessment of guideline-driven diagnostic performance. The results demonstrate that both guidelines offer similar accuracy in identifying severe disease stages in IPMNs, with certain clinical markers—such as jaundice, solid mass presence, and an increase in CA 19-9 levels—being pivotal in predicting the need for surgical intervention. This study concludes that while both guidelines provide valuable frameworks for IPMN management, there is an inherent need for further research to refine these protocols and improve patient-specific treatment strategies. This research contributes to the ongoing discourse on optimizing diagnostic and treatment paradigms for pancreatic cystic neoplasms, aiming to enhance clinical outcomes and patient care in this challenging medical field.

## 1. Introduction

Pancreatic cystic neoplasms (PCNs) are a heterogeneous collection of lesions characterized by the presence of cystic fluid-filled sacs inside the pancreas. They are classified into four types: serous cystic neoplasms (SCNs), mucinous cystic neoplasms (MCNs), intraductal papillary mucinous neoplasms (IPMNs), and solid pseudopapillary neoplasms (SPNs) [1]. All groups of PCNs, but not SCNs, carry a varied risk of cancer. The chance of malignancy differs depending on the form of IPMN, with side-branch IPMNs having a lower likelihood of developing cancer compared to other types of IPMNs [2]. These lesions span a broad range of clinical entities. The malignant potential differs amongst the different forms of PCNs, thus requiring a comprehensive strategy to discriminate between them. This involves a combined assessment of clinical, radiological, and endoscopic ultrasonography (EUS) findings, preferably in a multidisciplinary team. The malignant potential of PCNs regarding risks associated with surgical intervention should be carefully evaluated, therefore providing the most appropriate management (i.e., nonoperative or operative) for each patient.

It is crucial to acknowledge that IPMNs are a distinct subgroup of PCNs. These neoplasms are defined by the excessive growth of cells that produce mucin, a gel-like substance, within either the main pancreatic duct or its branches [3]. Lately, there has been an increasing acknowledgment of IPMNs attributed to advancements in imaging technology and an increasing awareness among doctors and a notable increase in the occurrence of IPMNs both in Europe and globally. The precise prevalence of IPMNs remains uncertain due to the predominance of asymptomatic cases. Nevertheless, studies suggest that the prevalence of this phenomenon is roughly 2.6–13.5%, with individuals commonly experiencing it throughout their fifth to seventh decade of life [4]. The condition is distinguished by the presence of papillary growths within the pancreatic ductal system, accompanied by the release of thick mucus. These growths have a potential risk of developing into malignancies [5]. The issue of whether surgical resection is beneficial for patients with IPMN continues to pose a significant surgical obstacle in contemporary times. Early identification, the evaluation of possible harm, and focused monitoring are essential in improving patient survival and quality of life because of the possibility of IPMNs progressing to invasive pancreatic cancer. 

To address this challenge, numerous international expert committees have developed evidence-based guidelines to aid physicians in risk stratification, monitoring, and therapeutic decision making. There are two prominent sets of guidelines that have become significant sources of reference in the treatment of IPMNs: the Fukuoka consensus guidelines (FCGs) and the European Evidence-based Guidelines for pancreatic cystic neoplasms (EEBGPCNs). The FCGs outlined the identification of high-risk stigmata and worrying traits [6]. Specifically, it is advised to promptly perform resection in cases with high-risk stigmata, whereas a conservative approach is advocated when concerning features are present. The FCGs underwent minor revisions and updates in 2017, in conjunction with the incorporation of fresh literature findings [7]. The presence of three characteristics was identified, as high-risk stigmata signify a conclusive need for surgical intervention. Moreover, the identification of any of the nine concerning characteristics is regarded as an indication for the utilization of EUS, which provided a reaction to the study conducted by Tanaka et al. [6]. In 2013, the European Study Group on Cystic Tumors of the Pancreas offered the Expert Consensus, which established a definitive differentiation between absolute and relative justifications for surgical intervention. Additionally, the group outlined the recommended intervals for surveillance, including biannual assessments during the initial year and annual evaluations after that [8]. In 2018, the European Consensus underwent revisions and introduced the EEBGPCNs. This represented the initial set of recommendations based on evidence for the management of PCNs [2]. The guidelines implemented novel relative indicators for resections: the observed growth rate exceeding 5 mm per year, the development of diabetes mellitus (DM), and severe pancreatitis because of IPMNs. Considering the possible variations in epidemiology, pathology, and the clinical manifestation of pancreatic cystic neoplasms, particularly IPMNs, it is imperative to conduct a comprehensive evaluation and comparison of the diagnostic criteria and management recommendations specified in these guidelines across heterogeneous patient cohorts. 

A retrospective study [9] was conducted on 68 patients with IPMNs who went through surgery at the Clinical Center of Serbia between January 2012 and December 2020. The study contrasted the diagnostic efficacy of the EEBGPCNs and FCGs to detect high-grade dysplasia/invasive carcinoma (HGD/IC) in IPMNs. The study found that the comparability of both recommendations in detecting HGD/IC in IPMNs is evident. However, there were statistically significant disparities between the low-grade dysplasia (LGD) and HGD/IC groups in terms of the absolute and relative indications for surgery as per the EEBGPCNs. The absolute indications were found to be superior to the relative indications in identifying HGD/IC in IPMNs. Therefore, the study suggests that the EEBGPCNs may be more useful than the Fukuoka consensus guidelines in identifying IPMNs that require resection. However, bearing in mind the relatively small number of samples and the fact that this study was single-centered, definite conclusions were not possible. The current study aims to assess the diagnostic precision and clinical significance of several sets of guidelines in separate geographic regions by examining patients with IPMNs in the centers of Belgrade and Ljubljana to equip physicians with evidence-based resources that could further enhance patient care and the global understanding of IPMN management.

## 2. Materials and Methods

### 2.1. Study Population

A total of 113 patients with IPMNs who completed surgery were retrospectively examined and categorized according to the two chosen guidelines. Malignancy was characterized as HGD and IC. This retrospective study included all patients who underwent any kind of pancreatic resection (i.e., cephalic pancreatoduodenectomy, distal pancreatectomy, or total pancreatectomy) with histologically proven IPMNs.

The samples from the University Medical Center Ljubljana, the Clinical Department of Abdominal Surgery, were obtained from January 2017 to December 2022. The samples from the Clinical Center of Serbia, Belgrade, Serbia were collected between 2012 and 2023 (the first 68 patients were already analyzed, and the results were published [9], and an additional 8 cases were collected between December 2020 and May 2023). All patients underwent pancreatic resection (cephalic pancreatoduodenectomy, distal pancreatectomy, or total pancreatectomy). This study collected extensive data on the participants, including their age at the moment of surgery, sex, the presence of symptoms (specifically jaundice and previous episodes of acute pancreatitis), the presence of DM, the occurrence of new-onset DM, initial CA 19-9 serum levels, imaging studies, and surgical and pathologic indications. The preoperative imaging diagnosis was established with the use of various diagnostic techniques, including EUS, multi-sliced computed tomography (MSCT), magnetic resonance imaging (MRI), magnetic resonance cholangiopancreatography (MRCP), and endoscopic retrograde cholangiopancreatography (ERCP), following the suction of pure pancreatic juice. The surgical pathology specimens underwent evaluation by pathologists with extensive expertise. The patients were further categorized according to the presence of absolute indications, such as a positive cytology for malignancy or high-grade dysplasia, the presence of a solid mass, jaundice, as well as relative indications, including an enhancing mural nodule larger than 5 mm, a dilation of the main pancreatic duct equal to or greater than 10 mm, a growth rate of 5 mm or more per year, elevated serum CA 19-9 levels exceeding 37 U/mL, a main pancreatic duct dilation ranging from 5 to 9.9 mm, a cyst diameter equal to or greater than 40 mm, new-onset diabetes mellitus, acute pancreatitis, and an enhancing mural nodule smaller than 5 mm for resection. These stratifications were performed according to the guidelines provided by the EEBGPCNs. The categorization also encompassed the use of the FCGs, specifically referring to high-risk stigmata and worrisome features. The patients were classified into different categories, namely benign, LGD, and HGD/IC, depending on pathological results indicating the severity of their illness. Our investigation involved comparing demographic data, radiological findings, and clinical characteristics across two groups. The study protocol was approved by the Ethical boards of the Clinical Center of Serbia and the School of Medicine, the University of Belgrade (no. 1322/11-8). 

### 2.2. Statistical Analysis

The numerical data were reported as means together with their corresponding standard deviations. Student’s *t*-test was used to evaluate the differences in demographic data and clinical characteristics among the groups. Categorical data are displayed as absolute values, accompanied by their corresponding percentages. The chi-square test was used to assess differences in demographic data and clinical characteristics across groups for categorical variables, or Fisher’s exact test was used. The performance of the European evidence-based guidelines and the Fukuoka consensus guidelines in detecting HGD/IC in pancreatic cystic neoplasms was evaluated using sensitivity, specificity, positive predictive values (PPVs), and negative predictive values (NPVs). Univariate and multivariate logistic regression models were employed to determine the primary predictors of high-grade dysplasia/invasive cancer. The results are displayed as odds ratios together with their matching 95% confidence intervals. A receiver operating characteristic (ROC) curve analysis was implemented to test the diagnostic accuracy of the calculated European evidence-based guidelines’ and Fukuoka consensus guidelines’ summary scores in HGD/IC (vs. LGD) or IC (vs. HGD) patients’ status prediction. The Youden index was computed to facilitate the determination of cut-off values for summary scores.

The significance level for all the studies was established at 0.05. The statistical evaluation was conducted employing the IBM SPSS statistical software (SPSS for Windows, release 26.0, SPSS, Chicago, IL, USA).

## 3. Results

### 3.1. Slovenian Patients: Results

Table 1 displays the clinical features of the study population for IPMNs. The average age was 70.8 ± 10.9, with 62.2% of the participants being male. The majority of the lesions were found in the pancreatic head, specifically 89.2% of them. Main duct lesions were present in 51.5% of the participants, branch duct lesions were present in 63.6% of the participants, and mixed types in 27.3% of the participants. High-grade lesions were present in 21 (56.8%) patients. Among the high-grade lesions, 18 (85.7%) were carcinoma.

Table 2 displays the criteria for surgical removal based on the Fukuoka consensus recommendations and the EEBGPCNs for pancreatic cystic neoplasms, categorized by the ultimate surgical pathology findings. Based on the Fukuoka consensus, there was a notable difference in the occurrence of obstructive jaundice as an indication for resection between the LGD and HGD/IC groups, which are considered high-risk stigmata. Significant differences were seen in the size (3 cm), primary pancreatic duct diameter (5–9 mm), and high CA 19-9 levels between the LGD and HGD/IC groups, which are worrisome features indicating the need for resection. 

The EEBGPCNs found substantial differences in solid mass and jaundice in the LGD and HGD/IC groups. Furthermore, there was a notable disparity in the occurrence rate of at least one clear indication for resection between the LGD and HGD/IC groups. Within the context of relative indications, there was a significant difference in the levels of serum CA 19-9 (>37 U/mL) among the LGD and HGD/IC groups.

Table 3 displays the Fukuoka consensus recommendations and the diagnostic performance of resection indications of the EEBGPCNs. The Fukuoka consensus recommendation to diagnose HGD/IC in all IPMN patients had 66.7% sensitivity, 50% specificity, 53.3% PPV, and 63.6% NPV for at least one high-risk stigmata. Table 3 also indicates the Fukuoka consensus recommendations’ diagnostic performance of at least one high-risk stigmata and one worrying feature for resection (61.9%, 50.0%, 61.9%, and 50%). The diagnostic efficacy of at least one worrisome feature is not shown, because all patients have at least one. 

Using the final surgical pathology, Table 3 displays the diagnostic performance of resection indications according to European evidence-based recommendations divided by all IPMN patients. The European evidence-based guidelines for recognizing HGD/IC in all IPMN patients had 90.5% sensitivity, 50% specificity, 70.4% PPV, and 80% NPV for at least one absolute indication for resection. All IPMNs had an 81.0%, a 62.5%, a 73.9%, and a 71.4% diagnostic performance for at least one absolute and relative resection indication. Elevated serum CA 19-9 levels had a 66.7%, an 87.5%, and a 66.7% diagnostic performance.

### 3.2. Comparison of Slovenian and Serbian Participants 

Table 4 shows the IPMN study population’s clinical characteristics by center (Belgrade and Ljubljana) outcomes. The mean age in the Belgrade group was 60.8 ± 10.5 and 70.8 ± 10.9 in the Ljubljana group, and patients in the Ljubljana group were significantly older (*p* < 0.001). A higher frequency of male patients was present in the Ljubljana group (62.2%vs. 52.6%), without a significant difference. Pancreatic head lesions predominated at the pancreatic head (61.8% vs. 89.2%), and two location tumors were present in the Belgrade group (13.2 vs. 0). 

Among the Belgrade group, high-grade lesions were present in 54 (71.1%) patients vs. the 21 (56.8%) patients in the Ljubljana group, without significant differences. Among the high-grade lesions, 40 (74.1%) were carcinoma in the Belgrade group and 18 (85.7) in the Ljubljana group.

An assessment of a preoperative evaluation and pathology report using these guidelines for the first 68 patients is presented in Djordjevic et al. [9].

### 3.3. Patients from Both Centers: Results

Table 5 displays the criteria for surgical removal based on the Fukuoka consensus recommendations and the EEBGPCNs for pancreatic cystic neoplasms, categorized by the final surgical pathology results. According to the Fukuoka consensus, there was a substantial difference in obstructive jaundice between the LGD and HGD/IC groups, which are considered high-risk stigmata for resection. Furthermore, the occurrence rate of at least one high-risk stigmata indicating resection was greater in the group with HGD/IC. Significant differences were observed in higher CA 19-9 levels, thicker and enhancing cyst walls, and lymphadenopathy between the LGD and HGD/IC groups, which are worrisome features indicating the need for resection. 

The EEBGPCNs observed substantial differences in solid mass and jaundice between the LGD and HGD/IC groups. Furthermore, there was a notable disparity in the occurrence rate of at least one clear indication for resection between the LGD and HGD/IC groups. Within the context of relative indications, there was a significant difference in the levels of serum CA 19-9 (>37 U/mL) between the LGD group and the HGD/IC group.

Table 6 displays the diagnostic accuracy of indications for resection based on the Fukuoka consensus guidelines and the EEBGPCNs. The Fukuoka consensus guidelines were used to determine the sensitivity, PPV, specificity, NPV, and precision for identifying HGD/IC in patients with IPMNs. The results show that the sensitivity, PPV, specificity, NPV, and accuracy for detecting at least one high-risk stigmata presence were 69.3%, 57.9%, 76.5%, and 48.9%, respectively. Additionally, the diagnostic success rate of at least one absolute and one relative indication for resection, according to the Fukuoka consensus guidelines, was found to be 68.0%, 57.9%, 76.1%, and 47.8%. 

Table 6 presents the diagnostic performance of indications for resection based on the final histological results, as determined by all patients with IPMNs according to the EEBGPCNs. The sensitivity, PPV, specificity, NPV, and accuracy for identifying HGD or IC in all patients with IPMNs according to European evidence-based recommendations were 85.3%, 47.4%, 76.2%, and 62.1%, respectively. The diagnosis accuracy for at least one absolute and at least one relative indication for surgical removal across all IPMNs was 82.7%, 55.3%, 78.5%, and 61.8%, respectively. Table 6 displays the diagnostic accuracy of a minimum of one relative indication and either a minimum of one absolute indication or one relative indication for resection, as outlined in the European evidence-based guidelines. Furthermore, this study presents the diagnostic accuracy of elevated serum CA 19-9 levels, which were found to be 69.3%, 89.5%, 92.9%, and 59.6%.

Table 7 displays the use of univariate and multivariate logistic regression to determine the primary predictors of HGD/IC. Initially, the Fukuoka characteristics were analyzed as indicators of HGD/IC. During the multivariate logistic regression analysis, it was found that both obstructive jaundice and CA 19-9 levels greater than 37 U/mL were independent and significant predictors of invasive IPMNs. The odds ratio for obstructive jaundice was 6.6, with a 95% confidence interval from 2.1 to 20.4, while the odds ratio for CA 19-9 levels greater than 37 U/mL was 29.9, with a 95% confidence interval from 7.8 to 114.5.

The EEBGPCN characteristics were analyzed to determine their ability to predict high-grade dysplasia or invasive carcinoma in the multivariate logistic regression analysis. It was found that solid mass and CA 19-9 levels greater than 37 U/mL were independent predictors of invasive IPMNs, with odds ratios of 5.1 (95% confidence interval of 1.6–16.6) and 15.1 (95% confidence interval of 4.5–51.3), respectively.

**Figure 1:**
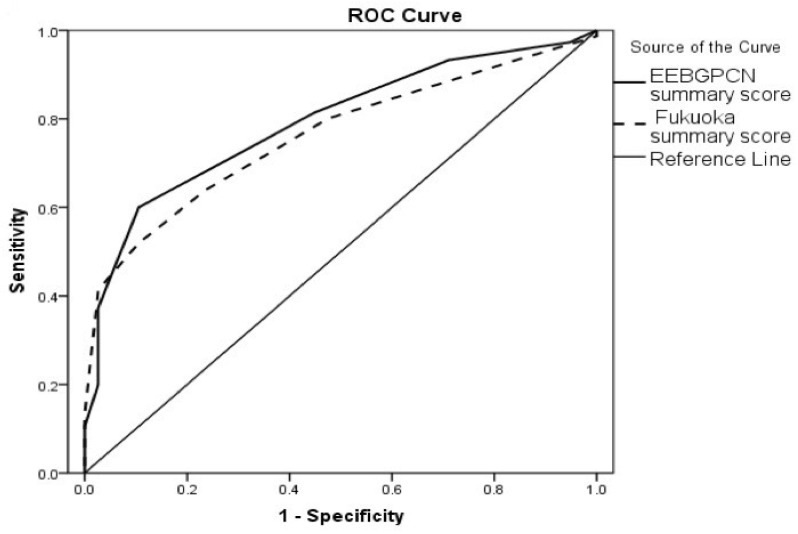
ROC analysis for diagnostic accuracy of EEBGPCNs and Fukuoka summary score estimations for HGD/IC (vs. LGD) patients’ status predictions: LGD—low-grade dysplasia, HGD—high-grade dysplasia, IC—invasive carcinoma.

To test diagnostic accuracy in HGD/IC status predictions of the newly calculated summary scores, an ROC analysis was implemented. The results of this part of the analysis are presented in Figure 1 and Table 8, while cut-off values with corresponding sensitivities and specificities produced from the ROC curves are presented in Table 9.

Both scores showed good diagnostic accuracy in HGD/IC vs. LGD patients’ status predictions (AUCs > 0.700), with a slightly higher EEBGPCN summary score accuracy. 

## 4. Discussion

The FCGs represent a notable progression in the management of IPMNs. These guidelines were established by a panel of international experts in Fukuoka, Japan, offering a comprehensive framework for risk stratification, diagnostic assessment, and therapeutic decision making in patients diagnosed with IPMNs [6,7]. The guidelines encompass a multidisciplinary approach that integrates radiographic, clinical, and histological criteria. The incorporation of this approach facilitates the evaluation of the potential for malignant alterations, hence enabling the customization of patient care. To assess the likelihood of malignancy, the guidelines incorporate the “worrisome features” and “high-risk stigmata” identified through the application of imaging modalities and endoscopic evaluation. The aforementioned criteria encompass multiple features, such as mural nodules, the extent of cystic lesions, the existence of obstructive jaundice, and the existence of enhancing solid components. The reality of these entities necessitates further investigation and, when appropriate, consideration of surgical removal as a method to decrease the risk of malignant progression. However, the implementation of the Fukuoka consensus guidelines is not devoid of challenges and limitations. The EEBGPCNs represent a significant scholarly addition to the field of managing IPMNs and other pancreatic cystic diseases within the European setting. These guidelines have been formulated through a collective endeavor involving esteemed European experts [2,8]. They provide a strong and empirically supported structure to assist physicians in conducting risk assessments, diagnostic evaluations, and making therapy decisions. They exhibit a distinct European-centric perspective, setting them apart from other established guidelines, such as the Fukuoka consensus guidelines and the guidelines provided by the American Gastroenterological Association (AGA), and providing absolute and relative indications for resection. Nevertheless, it is important to recognize certain restrictions that arise while utilizing the EEBGPCNs in the management of IPMNs. 

In the current investigation, the frequency of HGD/IC was determined to be 71.1% in the Belgrade group and 56.8% in the Ljubljana group. Despite being comparatively higher than in previous research [10,11], the observed percentage nevertheless fell within the range reported in the recent review paper that comprehensively outlines the latest breakthroughs in the identification and management of this matter [12]. In addition, it is worth noting that the age at which IPMN is typically diagnosed and the observed distribution of genders among patients align with important demographic characteristics.

The examination of the two cohorts, Serbian and Slovenian, highlights the intricacies associated with the use of comprehensive diagnostic criteria across heterogeneous populations [9]. Both groups emphasize that obstructive jaundice and elevated CA 19-9 levels are important indicators of HGD/IC in IPMNs, stressing the importance of these markers in guiding treatment options. Nevertheless, the cohort from Slovenia presented a more extensive range of characteristics, specifically tumor size and major pancreatic duct size, which were identified as relevant markers according to the Fukuoka consensus. This observation implies that Slovenian patients diagnosed with IPMNs may exhibit a broader range of clinical characteristics in comparison to their Serbian counterparts, hence requiring a comprehensive diagnostic strategy that considers several factors. When we examine diagnostic performance, the European recommendations have a higher sensitivity in Slovenia (90.5%) than in Serbia (82.0%). Clinical diagnostics rely on sensitivity to accurately identify illness patients. The European criteria seem to detect true HGD/IC cases better in Slovenian patients than in Serbian patients. Disease manifestation, genetics, environmental circumstances, or local medical procedures during preliminary diagnosis may cause such a disparity. The Fukuoka consensus indicated a greater PPV in Serbia (81.0%) than in Slovenia (50.0%). Such a disparity in PPV suggests that the Fukuoka consensus may better reflect the Serbian population or clinical landscape. There is a notable degree of heterogeneity in the implementation of guideline recommendations in real-life scenarios due to the disparities in distinct recommendations and practice patterns. The latest analysis indicates that there exists a significant potential for enhancing the awareness of the efficient dissemination of guidelines among healthcare providers in various practice settings [13]. The data presented underscore the significance of these enhancements for future endeavors. The serum marker CA 19-9 is of significant importance in both cohorts; nevertheless, its performance varies. Within the Serbian population, the remarkably high PPV of 94.6% indicates that an elevated CA 19-9 level serves as an exceedingly dependable indicator of HGD/IC. In contrast, the Slovenian cohort demonstrates a PPV of 87.5% for CA 19-9. Although both values exhibit commendable levels, the marginal decrease shown in the Slovenian cohort suggests that additional confounding factors may have an impact on the increase in CA 19-9 levels. The findings presented align with those reported in the study conducted on a cohort of 367 patients with surgically confirmed IPMNs at the Seoul National University Hospital. A preoperative assessment of serum tumor markers was conducted, revealing that patients with malignant IPMNs exhibited a notably elevated serum CA19-9 level. This finding demonstrates the diagnostic efficacy of serum CA 19-9 in predicting the presence of malignancy [14].

The new guidelines of the FCGs delineate three variables as high-risk stigmata and are deemed absolute surgical indications. Additionally, worrisome features are defined as an indication for EUS and FNA/FNC. The study confirmed that high-risk stigmata could identify patients with HGD/IC in IPMNs. Among the characteristics examined, it was shown that obstructive jaundice was strongly associated with a greater incidence of HGD/IC among all instances of IPMNs. The investigation, carried out on 230 patients with IPMNs [10], revealed the significance of this metric. The current study did not establish the presence of a specific worrisome feature as a parameter for identifying HGD/IC. However, there were notable differences in the elevated serum CA 19-9 levels, which confirmed the results of our earlier study [9]. However, the present study identified two new worrisome features, a thickened and enhancing cyst wall and lymphadenopathy, as significantly different between the LGD and HGD/IC groups. These findings suggest that these features may have the potential to identify HGD/IC patients in IPMNs. 

A recent study recruited 115 IPMN patients from six locations who had EUS within three months of surgery. EUS identified benign and malignant IPMNs, and the random forest forecasting model was able to accurately predict mural nodule IPMNs [15]. A recent study comprising 131 retrospective and 53 prospective patients, all of whom were histologically confirmed to have IPMNs, revealed that the circulating cytokine score (CCS), along with the existence of solid components and an MPD dilatation of 10 mm or more, were identified as key variables for determining the malignancy of IPMNs [16].

The present study found that jaundice and the appearance of a solid mass were strongly correlated with malignancy and were identified as reliable indicators of IPMNs with HGD/IC. These findings correspond with existing scientific sources [11,17,18] and confirm the results of our previous study [9]. On the other hand, the present study did not find the presence of mural nodes to be significantly associated with HGD/IC. However, the systematic review by Ohno et al. [19] identified a significant association between the presence of mural nodules that were ≥5 mm in diameter or solid components with a contrast enhancement and the diagnosis of HGD/IC. Keeping in mind the absence of a well-defined distinction between mural nodes and solid components in preoperative IPMN imaging and hence challenges in their differentiation, the authors emphasized the importance of incorporating both parameters in high-risk findings in IPMN clinical management [19]. 

The current study validated the use of elevated blood CA 19-9 levels as predictive markers for HGD/IC. These criteria, which have been recently implemented and are considered relative indications for resection based on the EEBGPCNs, are effective in identifying patients at risk of developing advanced stages of IPMNs. The present study proposes the inclusion of CA 19-9 as a beneficial adjunct in the therapy of IPMNs. 

A recent study by Lin at al., including an IPMN cohort of 186 patients, was examined. The investigators studied many factors such as tumor size, location, MPD diameter, the presence of a mural nodule, and abrupt changes in the caliber of the main pancreatic duct with distal pancreatic atrophy. These factors were evaluated using magnetic resonance imaging [20]. Aside from collecting demographic information, the study also acquired serum CA 19-9 and carcinoembryonic antigen (CEA) results. In the medical field, the classification of IPMNs as malignant has traditionally been based on the detection of HGD and concurrent IC. The findings of the study indicate that the detection of pancreatic atrophy by MRI imaging can assist healthcare professionals in improving the precision of diagnosing malignant IPMNs or invasive carcinoma, as well as in determining the most suitable treatment approach. Furthermore, the research findings indicate that incorporating risk variables, such as the dimensions of the tumor, the existence of a mural nodule, and increased levels of tumor biomarkers, such as CA 19-9 and CEA, could potentially assist in informing decisions on monitoring, surgical procedures, or alternative therapies for individuals diagnosed with IPMNs. 

Like all clinical guidelines, there is the potential for both false positives and false negatives, leading to the potential for the overdiagnosis or underdiagnosis of patients. The values of PPV and NPV of the FCGs (one high-risk stigmata) and the EEBGPCNs (one absolute indication) for HGD/IC were 76.5%, 48.9%, 76.2%, and 62.1%, respectively, suggesting similar predictive values of both guidelines, with the EEBGPCNs having a better NPV. The multivariate analysis revealed jaundice, solid mass, and elevated CA 19-9 as associated with HGD/IC in patients with IPMNs. In a similar observational study conducted on 138 surgically treated patients, the 2006 Sendai and 2012 Fukuoka guidelines for managing IPMNs were found to be useful in identifying significant factors associated with HGD or IC in IPMN patients. A multivariate analysis showed that the existence of jaundice, a tumor size of 3 cm or larger, the presence of a mural nodule on imaging, or being younger than 65 years old were all significant factors associated with HGD or IC in IPMN patients [21]. The systematic review conducted by Srinivasan et al. assessed the clinical usefulness and accuracy of the Sendai and Fukuoka consensus guidelines in managing IPMNs of the pancreas. The evaluation included the analysis of 22 retrospective studies [22]. The review presented a concise overview of the clinicopathological characteristics, PPV, and NPV of the guidelines. The outcomes of the investigations varied significantly, with certain studies reporting NPVs ranging from 89% to 100%, while others only identified an overall of four cases of invasive FCG IPMNs. The possibility for discrepancy in risk assessment consistency can be influenced by the heterogeneity in the interpretation of imaging parameters among different radiologists. Moreover, it is important to acknowledge that the current guidelines may not comprehensively address the diverse uncommon manifestations of IPMNs or the less commonly observed clinical scenarios. Consequently, the feasibility and applicability of these methods may be limited in some clinical scenarios. Furthermore, given the ever-evolving nature of medical knowledge, the recommendations must undergo periodic changes to ensure the inclusion of the most up-to-date information and advancements in the realm of IPMN research and healthcare. More research is also needed to find preoperative criteria for assessing the prognosis of all types of pancreatic cysts and carcinoma, as well as risk factors and illness molecular foundations. A lot of research has been conducted on the various preoperative variables for evaluating the prognosis of pancreatic cancer [23,24]. Similarly, knowledge of risk factors associated with pancreatic cancer has recently increased [25,26,27], as has knowledge of the molecular basis of cancer, opening up new avenues for innovative and more efficient potential therapeutic targets for cancer as a whole [28,29,30]. Recent review papers emphasized certain genetic alterations, as well as the cyst fluid analysis and cytopathology of new molecular markers, laboratory tests, and imaging tools, for the successful diagnosis and management of IPMNs [31].

Recent Kyoto guidelines confirmed the need for management improvement identified in the present study. They represented a significant update in the management of IPMNs by integrating evidence-based recommendations, refining risk assessment strategies, and addressing key clinical questions in a more structured and comprehensive manner [32]. These guidelines propose a new management algorithm, which includes the assessment of high-risk stigmata and worrisome features based on imaging findings from EUS and a cytological analysis from the EUS-guided fine needle aspiration technique. This updated algorithm reflects advancements in diagnostic modalities and emphasizes the importance of these tools in risk assessment. The guidelines address the question of lifetime surveillance for small IPMNs and recommend the following options: “stop surveillance” or “continue surveillance for possible development of concomitant pancreatic ductal adenocarcinoma” for small unchanged branch duct IPMNs (BD-IPMNs) after 5 years of surveillance. The new guidelines prioritize the examination of pathological features and the study of molecular markers in cyst fluids [32]. 

Our research does certainly have a few limitations. First, because this is a retrospective study, it is unavoidable that there will be some selection bias. However, to strengthen the generalizability of our findings, we gathered data from two different centers. Second, the size of our sample was limited, especially when centers were observed separately. Lastly, this study does not contain data on follow-ups of the individuals without surgery, i.e., whether they developed cancer and, if so, after what period of time. 

## 5. Conclusions

This study offers vital insights into the efficacy of the FCGs and EEBGPCNs in treating IPMNs across various centers. Based on the data collected from two European facilities, this study suggests that both guidelines have a comparable capacity to accurately diagnose HGD/IC in PCNs. The discovered discrepancies emphasize the need to validate standards across communities. The observed interplay of sensitivity, specificity, and predictive values across the two populations emphasizes the need for tailored patient management. It has been confirmed that high-risk stigmata indications are more effective than worrisome features in the identification of HGD/IC in IPMNs, while the EEBGPCNs’ absolute indications exhibit superior performance in this regard. Elevated blood CA 19-9 levels together with a thickened and enhancing cyst wall and lymphadenopathy have been recognized as a significant relative criterion for surgical procedure utilization in IPMN management across all IPMN subtypes. Further research should be focused on improving the accuracy of these guidelines and identifying improved ways to manage patients with IPMNs. Nevertheless, it is imperative to acknowledge the limitations of their utilization, and it is essential to continue doing research and fostering interdisciplinary collaboration to enhance and optimize the management strategies for IPMNs in clinical settings.

## Figures and Tables

**Table 1:** Demographic data of patients with IPMNs.

		Ljubljana *n* = 37
Age, mean ± sd		70.8 ± 10.9
Sex, *n* (%)	Female	14 (37.8)
	Male	23 (62.2)
Location, *n* (%)	Head	33 (89.2)
	Body	1 (2.7)
	Tail	3 (8.1)
Main duct, *n* (%)		12 (32.43)
Branch duct, *n* (%)		16 (43.24)
Mixed type, *n* (%)		9 (24.3)
Grade, *n* (%)	Low/moderate	16 (43.2)
	High grade	21 (56.8)
High grade, *n* (%)	High grade	3 (14.3)
	Carcinoma	18 (85.7)

**Table 2:** Resection indications for pancreatic cystic neoplasms according to Fukuoka and EEBGPCN consensus standards.

	LGD *n* = 16	HGD/IC *n* = 21	*p*
Fukuoka consensus guidelines			
Obstructive jaundice	3 (18.8)	13 (61.9)	0.009
Enhancing solid component > 5 mm	1 (6.3)	1 (4.8)	1.000
Main pancreatic duct 10 mm	5 (31.3)	2 (9.5)	0.202
At least one high-risk stigmata indication for resection	8 (50)	14 (66.7)	0.306
Size, 3 cm	11 (68.8)	6 (28.6)	0.015
Enhancing mural nodule, <5 mm	2 (12.5)	0 (0)	0.180
Thickened and enhancing cyst wall	3 (18.8)	0 (0)	0.072
Main pancreatic duct, 5–9 mm	5 (31.3)	15 (71.4)	0.015
Elevated CA 19-9	2 (12.5)	14 (66.7)	0.001
Cyst growth rate 5 mm in 2 years	5 (31.3)	1 (4.8)	0.066
Abrupt change in caliber of the pancreatic duct with distal pancreatic atrophy	0 (0)	0 (0)	NA
Lymphadenopathy	2 (12.5)	3 (14.3)	1.000
At least one worrisome feature	16 (100)	19 (90.5)	0.495
EEBGPCNs			
Positive cytology for malignancy/HGD	0 (0)	4 (19)	0.118
Solid mass	2 (12.5)	14 (66.7)	0.001
Jaundice	3 (18.8)	13 (61.9)	0.009
Enhancing mural nodule, >5 mm	0 (0)	1 (4.8)	1.000
MPD dilation, 10 mm	5 (31.3)	2 (9.5)	0.202
At least one absolute indication for resection	8 (50)	19 (90.5)	0.009
Grow rate, 5 mm/year	5 (31.3)	1 (4.8)	0.066
Increased levels of serum CA 19-9, >37 U/mL	2 (12.5)	14 (66.7)	0.001
MPD dilation between 5 and 9.9 mm	5 (31.3)	13 (61.9)	0.065
Cyst diameter, 40 mm	4 (25)	4 (19)	0.705
New-onset DM	0 (0)	1 (4.8)	1.000
Acute pancreatitis	2 (12.5)	3 (14.3)	1.000
Enhancing mural nodule, <5 mm	2 (12.5)	0 (0)	0.180
At least one relative indication for resection	12 (75)	19 (90.5)	0.371

Data are presented as *n* (%).

**Table 3:** Diagnostic performance of resection indications as per Fukuoka consensus guidelines and EEBGPCNs in Slovenian patients.

	Sn	Sp	PPV	NPV
**Fukuoka consensus**				
At least one high-risk stigmata	66.7%	50%	63.6%	53.3%
At least one high-risk stigmata and one worrisome feature	61.9%	50%	61.9%	50%
**EEBGPCNs**				
At least one absolute indication	90.5%	50%	70.4%	80%
At least one absolute and one relative indication	81.0%	62.5%	73.9%	71.4%
At least one relative indication	90.5%	25.0%	61.3%	66.7%
Increased levels of serum CA 19-9	66.7%	87.5%	87.5%	66.7%

**Table 4:** Demographic data of patients with IPMNs according to study group.

		Belgrade *n* = 76	Ljubljana *n* = 37	*p*
Age, mean ± sd		60.8 ± 10.5	70.8 ± 10.9	<0.001
Sex, *n* (%)	Female	36 (47.4)	14 (37.8)	0.338
	Male	40 (52.6)	23 (62.2)	
Location, *n* (%)	Head	47 (61.8)	33 (89.2)	0.011
	Body	12 (15.8)	1 (2.7)	
	Tail	7 (9.2)	3 (8.1)	
	Two locations	10 (13.2)	0 (0)	
Main duct, *n* (%)		12 (15.79)	12 (32.43)	0.024
Branch duct, *n* (%)		28 (36.84)	16 (43.24)	0.045
Mixed type, *n* (%)		36 (47.37)	9 (24.3)	0.060
Grade, *n* (%)	Low/moderate	22 (28.9)	16 (43.2)	0.131
	High grade	54 (71.1)	21 (56.8)	
High grade, *n* (%)	High grade	14 (25.9)	3 (14.3)	0.280
	Carcinoma	40 (74.1)	18 (85.7)	

**Table 5:** Criteria for surgical removal based on the recommendations for pancreatic cystic neoplasms of the Fukuoka consensus and the EEBGPCNs.

	LGD *n* = 38	HGD/IC *n* = 75	*p*
**Fukuoka consensus guidelines**			
Obstructive jaundice	7 (18.4)	45 (60)	<0.001
Enhancing solid component, >5 mm	4 (10.5)	18 (24)	0.087
Main pancreatic duct, 10 mm	8 (21.1)	18 (24)	0.725
At least one high-risk stigmata indication for resection	16 (42.1)	52 (69.3)	0.005
Size 3 cm	19 (50)	41 (54.7)	0.639
Enhancing mural nodule, <5 mm	11 (28.9)	24 (32)	0.740
Thickened and enhancing cyst wall	11 (28.9)	38 (50.7)	0.028
Main pancreatic duct, 5–9 mm	22 (57.9)	52 (69.3)	0.227
Elevated CA 19-9	3 (7.9)	54 (72)	<0.001
Cyst growth rate, 5 mm in 2 years	5 (13.2)	3 (4)	0.073
Abrupt change in caliber of the pancreatic duct with distal pancreatic atrophy	1 (2.6)	5 (6.7)	0.366
Lymphadenopathy	11 (28.9)	37 (49.3)	0.038
At least one worrisome feature	38 (100)	73 (97.3)	0.310
**EEBGPCNs**			
Positive cytology for malignancy/HGD	1 (2.6)	5 (6.8)	0.351
Solid mass	8 (21.1)	54 (72)	<0.001
Jaundice	7 (18.4)	42 (56.8)	<0.001
Enhancing mural nodule, >5 mm	3 (7.9)	14 (18.9)	0.124
MPD dilation, 10 mm	9 (23.7)	16 (21.6)	0.804
At least one absolute indication for resection	20 (52.6)	64 (85.3)	<0.001
Grow rate, 5 mm/year	5 (13.2)	2 (2.7)	0.029
Increased levels of serum CA 19-9, >37 U/mL	4 (10.5)	52 (69.3)	<0.001
MPD dilation between 5 and 9.9 mm	21 (55.3)	48 (64)	0.368
Cyst diameter, 40 mm	10 (26.3)	28 (37.3)	0.242
New-onset DM	3 (7.9)	7 (9.3)	0.799
Acute pancreatitis	4 (10.5)	10 (13.3)	0.669
Enhancing mural nodule, <5 mm	11 (28.9)	25 (33.3)	0.636
At least one relative indication for resection	33 (86.8)	71 (94.7)	0.147

Data are presented as *n* (%).

**Table 6:** The diagnostic performance of indications for resection, as per the Fukuoka consensus recommendations and the EEBGPCNs, evaluated based on the findings collected from both centers.

	Sn	Sp	PPV	NPV
**Fukuoka consensus**				
At least one high-risk stigmata	69.3%	57.9%	76.5%	48.9%
At least one high-risk stigmata and one worrisome feature	68%	57.9%	76.1%	47.8%
**EEBGPCNs**				
At least one absolute indication	85.3%	47.4%	76.2%	62.1%
At least one absolute and one relative indication	82.7%	55.3%	78.5%	61.8%
At least one relative indication	67.3%	94.7%	68.3%	55.7%
At least one absolute or one relative indication	98.6%	0%	67%	0%
Increased levels of serum CA 19-9	69.3%	89.5%	92.9%	59.6%

**Table 7:** Results of both univariate and multivariate logistic regression analyses, which were conducted to determine the most significant predictors of high-grade dysplasia/invasive cancer.

	Univariate				Multivariate			
	OR	95% C.I. for OR		*p*	OR	95% C.I. for OR		*p*
		Lower	Upper			Lower	Upper	
**FCG**								
Obstructive jaundice	6.643	2.591	17.028	<0.001	6.593	2.132	20.39	0.001
Enhancing solid component, >5 mm	2.684	0.838	8.594	0.096				
Main pancreatic duct, 10 mm	1.184	0.461	3.04	0.725				
Size 3 cm	1.206	0.552	2.635	0.639				
Enhancing mural nodule, <5 mm	1.155	0.492	2.709	0.74				
Thickened and enhancing cyst wall	2.521	1.094	5.807	0.03				
Main pancreatic duct, 5–9 mm	1.644	0.732	3.695	0.229				
Elevated CA 19-9	30	8.322	108.148	<0.001	29.855	7.787	114.466	<0.001
Cyst growth rate, 5 mm in 2 years	0.275	0.062	1.22	0.089				
Abrupt change in caliber of the pancreatic duct with distal pancreatic atrophy	2.643	0.298	23.466	0.383				
Lymphadenopathy	2.39	1.037	5.506	0.041				
**EEBGPCNs**								
Positive cytology for malignancy/HGD	2.721	0.306	24.165	0.369				
Solid mass	9.643	3.81	24.406	<0.001	5.132	1.591	16.552	0.006
Jaundice	5.812	2.27	14.885	<0.001	2.21	0.647	7.544	0.206
Enhancing mural nodule, >5 mm	2.722	0.731	10.137	0.135				
MPD dilation, 10 mm	0.889	0.351	2.254	0.804				
Grow rate, 5 mm/year	0.181	0.033	0.981	0.047				
Increased levels of serum CA 19-9, >37 U/mL	19.217	6.107	60.474	<0.001	15.112	4.45	51.314	<0.001
MPD dilation between 5 and 9.9 mm	1.439	0.65	3.185	0.369				
Cyst diameter, 40 mm	1.668	0.706	3.943	0.244				
New-onset DM	1.201	0.292	4.932	0.799				
Acute pancreatitis	1.308	0.382	4.481	0.669				
Enhancing mural nodule, <5 mm	1.227	0.525	2.871	0.637				

**Table 8:** The basic parameters of an ROC analysis for the diagnostic accuracy of EEBGPCN and Fukuoka summary scores in HGD/IC (vs. LGD) patients’ status predictions.

Test Result Variable(s)	HGD/IC (vs. LGD) Prediction				
	Area under the Curve (AUC)	Std. Error	*p*	Asymptotic 95% Confidence Interval	
				Lower Bound	Upper Bound
EEBGPCN summary score	0.792	0.043	<0.001	0.708	0.875
Fukuoka summary score	0.762	0.044	<0.001	0.675	0.848

LGD—low-grade dysplasia, HGD—high-grade dysplasia, IC—invasive carcinoma.

**Table 9:** Summary scores’ cut-off values as determined by the Youden index calculation method and corresponding sensitivities and specificities.

Parameter	HGD/IC (vs. LGD) Prediction			IC (vs. HGD) Prediction		
	Cut-Off Value	Sensitivity (%)	Specificity (%)	Cut-Off Value	Sensitivity (%)	Specificity (%)
EEBGPCN summary score	3.5	60.0	89.5	3.5	66.7	66.7
Fukuoka summary score	4.5	52.0	89.5	2.5	87.7	47.6

LGD—low-grade dysplasia, HGD—high-grade dysplasia, IC—invasive carcinoma.

## Data Availability

Data is available upon reasonable request.
